# Patient experiences of hydrodistension as a treatment for frozen shoulder: A longitudinal qualitative study

**DOI:** 10.1371/journal.pone.0304236

**Published:** 2024-06-14

**Authors:** Gareth Whelan, Gillian Yeowell, Chris Littlewood

**Affiliations:** 1 Extended Scope Practitioner (Upper Limb) York and Scarborough Teaching Hospitals NHS FT, United Kingdom; 2 Professor of Musculoskeletal Physiotherapy Health and Wellbeing, Manchester Metropolitan University, Manchester, United Kingdom; 3 Professor of Musculoskeletal Research, Edge Hill University, Ormskirk, United Kingdom; Universidad Santiago de Cali, COLOMBIA

## Abstract

**Background:**

Frozen shoulder is a condition associated with severe shoulder pain and loss of function impacting on a persons’ physical and mental health. Hydrodistension treatment that has been widely adopted within the UK National Health Service for the condition. However, evidence of clinical effectiveness and understanding of the patient experiences of this treatment are lacking. This study explored the experiences of people with a frozen shoulder who received hydrodistension treatment.

**Methods:**

A qualitative design with repeat semi-structured interviews was used to explore participants’ experiences of hydrodistension treatment. Participants were interviewed 2–4 weeks and again at 8–10 weeks after treatment. Interviews were audio-recorded and transcribed verbatim. Findings were analysed using an inductive thematic analysis framework. The study is reported in accordance with the consolidated criteria for reporting qualitative (COREQ) research.

**Results:**

15 participants were interviewed online or over the phone. Three themes were identified: ‘Preparing for and having a hydrodistension’, ‘Physiotherapy after hydrodistension’, and ‘Outcome of hydrodistension ‘. Participants believed hydrodistension would benefit them, was well tolerated by many, and the effects were apparent to most within the first week. Physiotherapy still seemed to be valued to support recovery beyond this timepoint, despite these early effects. Some participant’s experienced harms including severe procedural pain and blood sugar dysregulation.

**Conclusion:**

This is the first study to investigate the experiences of people who undergo hydrodistension for frozen shoulder. Hydrodistension appears an acceptable treatment to participants with a frozen shoulder, acceptability is enhanced through adequate shared decision making. Further high-quality research is required to understand the comparative effectiveness of hydrodistension as a treatment for frozen shoulder, including adverse events, and the benefit of treatment by a physiotherapist after hydrodistension.

## Introduction

Frozen shoulder is a condition associated with severe pain, sleep disturbance, and a loss of function in the affected arm [1]. These symptoms are persistent for many, lasting over 12 months with about 20% of people not fully recovering [2]. The persistent nature of symptoms has been reported to have a significant effect on physical and mental health with those affected reporting loss of independence, functional capability, and an altered sense of self [3, 4].

The pathophysiology of the condition is not fully understood [1], however it is characterised as an inflammatory mediated fibroproliferative disorder [5], causing the clinical symptoms of pain and stiffness. It is not known how or why people develop a frozen shoulder [6] however female sex [7], diabetes [8, 9], thyroid disorders [10], and genetic predisposition [11] are all considered risk factors.

In the United Kingdom (UK) there is no nationally accepted treatment and management pathway for people with frozen shoulder and patients may be offered a range of treatments including physiotherapy, corticosteroid injections, or referral for surgical care [12]. Within these varied treatment pathways, hydrodistension, an injection-based treatment, is becoming increasingly popular [12]. The treatment involves injecting a combination of local anaesthetic, corticosteroid, and sterile saline into the shoulder joint under image guidance [13]. The aim of the treatment is to deliver a therapeutic dose of corticosteroid and to inject a sufficient volume of fluid to achieve a distension of the shoulder joint capsule, this is usually greater than 20 millilitres (ml) but typically between 30ml and 40ml. The stated aim of the treatment is to reduce pain and improve function. The comparative effectiveness of hydrodistension to other treatments for people with frozen shoulder remain uncertain [14]. Despite this uncertainty the treatment is now offered widely in the UK National Health Service (NHS).

Previous studies have explored patient experiences of other treatments for frozen shoulder such as corticosteroid injection, physiotherapy, and surgery [15, 16]. However, there is an absence of research considering the patient experience of hydrodistension including why patients choose to have hydrodistension, the experience of the procedure, and their post-treatment response trajectory. Thus, this current study aimed to explore participants’ experience of hydrodistension as a treatment for frozen shoulder in order to inform shared decision making in clinical practise and identify evidence gaps requiring further exploration.

## Methods

A qualitative research design with repeat semi-structured interviews was undertaken to investigate the aim. This qualitative interview study is the first step in a planned multi-methods programme of research that is presented within a pragmatic framework. The study is reported in accordance with the consolidated criteria for reporting qualitative (COREQ) research [17]. Ethical approval was obtained on the 12^th of^ October 2022 from South West-Frenchay Research Ethics Committee (ref: 22/SW/0122) and the UK Health Research Authority (ref:307480).

We planned to recruit up to 20 participants with frozen shoulder who had elected to have hydrodistension treatment. A purposive sampling strategy was used, with the aid of a sampling frame, to ensure we captured participants with diverse experience of frozen shoulder. To ensure our sample was reflective of the frozen shoulder population we included more female than male participants [7], participants with type 1 and type 2 diabetes [8, 9] and participants at different stages of frozen shoulder (pain dominant, pain and stiffness, resolution phase) [18]. An iterative process drawing upon the concept of thematic saturation [19] was used to determine the final sample size. In practise this meant that once themes had been developed, and analysis of new data was generating no new themes, we considered that thematic saturation had been reached and recruitment ceased.

Participants were recruited from the musculoskeletal and physiotherapy departments of two participating NHS Trusts between 10^th^ November 2022 and 24^th^ February 2023. Participants were eligible for inclusion in the study if during their usual NHS care they were diagnosed with a frozen shoulder and they opted to undergo hydrodistension treatment. The diagnostic criteria clinicians used for frozen shoulder were: a loss of greater than half their passive range of external rotation on the symptomatic side compared with their asymptomatic side [20] in the presence of a normal plain film Xray and, in the case of traumatic onset, exclusion of rotator cuff pathology via ultrasound or MRI. Written consent to contact for eligible potential participants was obtained by clinicians working at the participating NHS trusts. Informed consent to participate was then obtained by audio recording or in writing by the lead researcher [GW] prior to participation.

One to one semi structured interviews were undertaken by the lead researcher with participants via Microsoft teams or telephone and digitally audio recorded. Each participant completed two interviews. The first interview took place two to four weeks after undergoing hydrodistension, the second interview took place eight to ten weeks after undergoing hydrodistension. Interviews lasted between 9 and 32 minutes. A topic guide (S1 Appendix) had been developed by the lead researcher with feedback from a patient and public involvement (PPI) group consisting of 5 members (3 female, 2 male) with experience of shoulder pain, frozen shoulder, and hydrodistension. The topic guide was used to direct the interviews. The interviews were conducted by the lead researcher, a male clinical-academic physiotherapist with qualitative research training who also performs hydrodistension within his NHS clinical role. The study participants were unknown to the interviewer. Participants were aware that the lead researcher was a clinical academic physiotherapist, but unaware that he delivered hydrodistension as part of his clinical role. Only the lead researcher had access to information that could identify individual participants during or after the interviews.

Data analysis was conducted using Braun and Clarke’s six stage model of thematic analysis [21]. Interviews were pseudonymized and transcribed verbatim by the lead researcher [GW] to facilitate the first stage of the model, data familiarisation. Transcripts were not returned to participants as no data quality issues were identified. The second stage of the model, assigning preliminary codes, was completed by the lead researcher. The data was read line by line to identify text relevant to the research question and preliminary codes were derived to summarise the data. Codes were recorded in a Microsoft excel (Excel for mac version16.78) spreadsheet. Codes were subsequently explored within and across the dataset and iteratively refined. Cognate codes were grouped, and sub themes developed (stage 3). Sub themes that were conceptually similar were grouped together and preliminary themes were generated from the data (stage 4). These themes and subthemes were then critically reviewed and refined by the research team (GW,GY,CL) (stage 5). Finally, the data analysis was reported within this paper (stage 6). Throughout the data analysis process preliminary sub-themes and themes were used inductively to refine the topic guide and explored in subsequent interviews. A summary of the themes were sent by email to participants for comment and member checking.

## Results

Eighteen people gave consent to be contacted for the study. Fifteen participants were interviewed. The reasons for non-inclusion of the other three participants were failure to respond to contact, contact made outside the time window for the first interview, and the decision to halt recruitment due to thematic saturation being achieved.

All participants interviewed for the study had experienced a hydrodistension as treatment for their frozen shoulder within the last four weeks of their first interview. Three participants had prior experience of the treatment, having received hydrodistension for treatment of previous frozen shoulder in their other shoulder. The participant characteristics are reported in Table 1.

**Table 1:** Participant characteristics.

Participant	Sex	Stage of frozen shoulder	Diabetes	Previous hydrodistension (contralateral arm)
**1**	Female	2	-	No
**2**	Male	2	-	No
**3**	Male	3	Type 2	No
**4**	Female	3	-	Yes
**5**	Female	2	Type 1	No
**6**	Male	2	-	Yes
**7**	Male	1	-	No
**8**	Male	2	Type 2	No
**9**	Female	2	-	No
**10**	Male	2	Type 1	No
**11**	Female	2	-	Yes
**12**	Female	2	-	No
**13**	Male	2	-	No
**14**	Female	2	-	No
**15**	Female	1	-	No

### Themes

**Fig 1:**
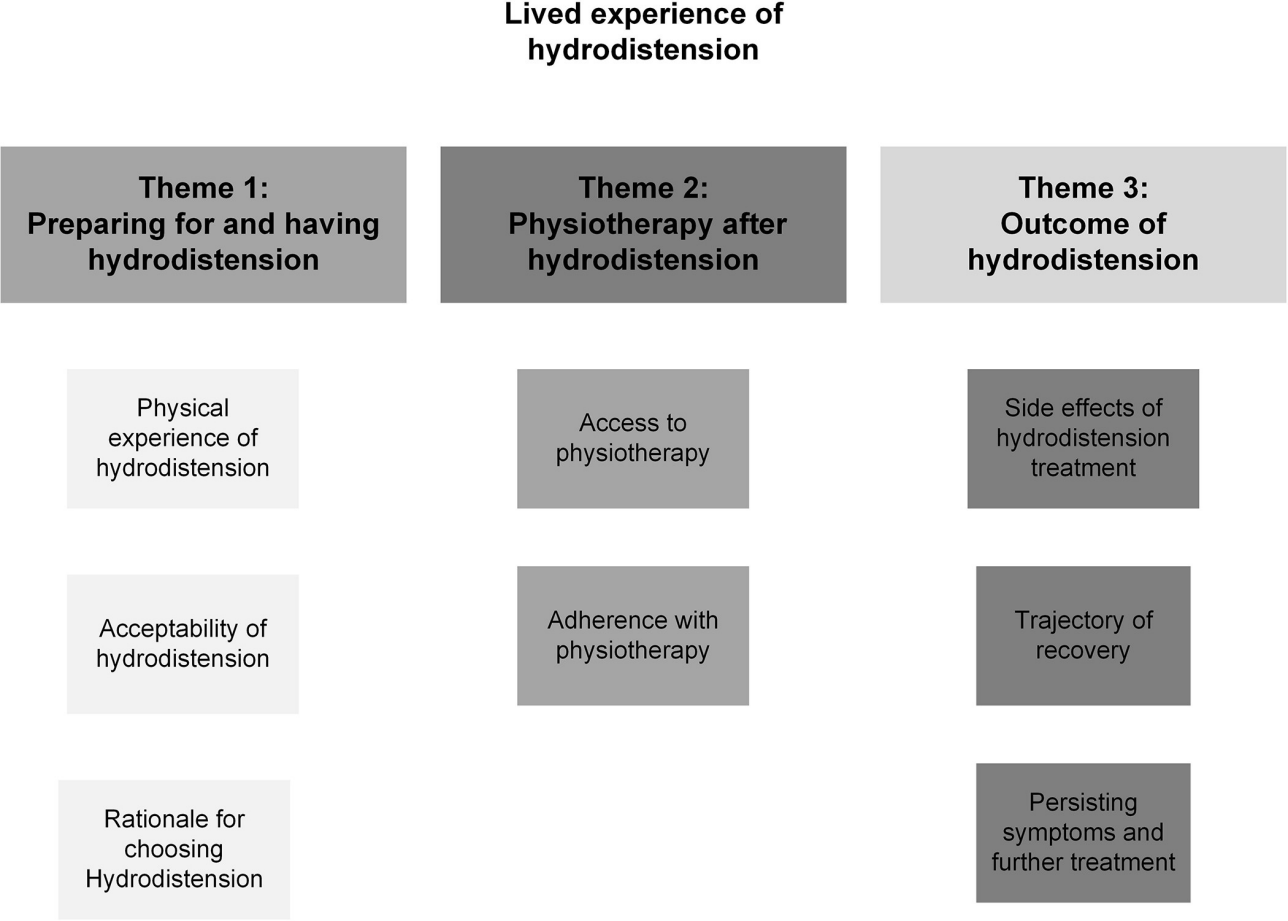
Themes and subthemes.

Three themes were identified from the data: ‘Preparing for and having hydrodistension’, ‘Physiotherapy after hydrodistension’, and ‘Outcome of hydrodistension’. Several subthemes were identified within each theme (Fig 1) and have been used to structure the results. Verbatim pseudonymised quotes have been used to support the themes.

#### Theme 1 ‘Preparing for and having hydrodistension’

‘Preparing for and having hydrodistension’ describes the factors influencing the decision to undergo hydrodistension, the experience of the treatment, and the acceptability of the treatment to a person with frozen shoulder.

#### Rationale for choosing hydrodistension

Participants described their rationale for choosing hydrodistension as a treatment for their frozen shoulder. This was grounded in their understanding of the condition and the physical effects they anticipated from the procedure.

“He [the physiotherapist] explained what it was and that it was to expand the shoulder muscle and… hopefully loosen it”.

Participant 13

“[The physiotherapist] described the outline of the procedure that it involved an injection into the shoulder of a combination of steroids and a saline solution to lift the tissue away from the bone and relieve movement”.

Participant 2

“That you get the injection and then inject the saline solution which will hopefully, hopefully open the joint up and allow it [to] move more”.

Participant 4

Participants reflected on their understanding of frozen shoulder as a condition characterised by stiffness and reduced mobility. This understanding underpinned their rationale for seeking a treatment to address these specific issues. The participants articulated their expectation that the treatment would have a specific physical effect on the soft tissues around the shoulder, variously characterising this as ‘opening up’ the joint or improving movement. Participants also reported the significant influence information provided by healthcare providers had on their understanding of the hydrodistension treatment. Participants made a reasoned choice to undergo hydrodistension based upon a collaborative decision making process with their healthcare provider. Both healthcare providers and participants appeared optimistic that the hydrodistension treatment would have an impact on the physical restriction being experienced by the participants in their shoulders.

Participants reported that part of their decision making process around proceeding with a hydrodistension involved rejecting a low volume intraarticular corticosteroid injection.

“Because the physio suggested that that would be more beneficial, and it would help me better. Because maybe the steroid injection if I had that, it would only come back again and I’d have to have another one done. Whereas this one might, she didn’t say it would, but she said it might help quicker”.

Participant 9

“And he [the physiotherapist] explained that this injection it was similar to the injection, the injection I had already had, but it’s much larger dose and it was an injection of an aquatic”.

Participant 13

Participants once again reported that information from healthcare providers had a significant influence on their decision making process. Participants reflected that conversations with their healthcare providers led them to believe that a hydrodistension was a ‘superior treatment’ to a ‘standard’ corticosteroid injection. Participants articulated this superiority in a number of ways. Participants mentioned concerns about potential reoccurrence with a standard injection and hydrodistension was perceived as offering a more lasting solution. It was also expressed by participants that hydrodistension had the potential to offer quicker relief of symptoms. Participants also framed hydrodistension as a superior treatment in terms of its pharmacological components suggesting that it may contain a larger dosage of active ingredient or that there was additional benefit from the addition of water based injectables. A rationalisation of these perceived differences between a ‘standard’ corticosteroid injection and hydrodistension facilitated participants to reject the standard corticosteroid injection in favour of hydrodistension and contributed significantly to their treatment decision.

Participants also reported that healthcare providers made a specific recommendation for the treatment.

“So when I saw the surgeon, he said, you know, he was like if it was my shoulder or my wife’s shoulder, I would definitely have hydrodistension”.

Participant 15

Participants demonstrated that alongside their understanding of frozen shoulder and hydrodistension being informed by healthcare providers, the recommendation of providers regarding treatment carried significant weight. This suggests that participants considered healthcare providers to have significant authority and expertise to be able to make sound judgements and recommendations. This trust in healthcare providers contributed to participants decisions to proceed with hydrodistension.

In summary participants made a decision to proceed with hydrodistension based on their understanding of frozen shoulder and how the treatment may positively affect their symptoms. They weighed up and rejected other treatments in favour of hydrodistension based on their perception that hydrodistension was a superior treatment. Participants knowledge was heavily informed by their healthcare provider, and there was evidence that participants had high levels of trust in provider recommendations and information.

#### Experience of hydrodistension

Participants described a range of experiences while undergoing the treatment.

“I was unaware, apart from the fact that I was laying on a table with people around me, I was unaware that I was having a treatment”.

Participant 2

“It [the hydrodistension] was done in no time at all and it didn’t hurt whatsoever”.

Participant 1

Some participants described the hydrodistension procedure as being pain free. One participant described being completely unaware that the treatment was taking place. Some participants report that they perceived the procedure to be done very quickly. This demonstrates that for some participants having a hydrodistension came without almost any burden.

Other participants reported that the procedure was associated with discomfort and pain.

“So, I was lying on my back my arm was by the side, and then it was sort of turned over. So, to be honest that wasn’t a particular… with my range of movement that… it was, it was uncomfortable”.

Participant 10

“I mean, it’s 5 minutes of quite extreme pain on the table, but that’s more…[it] wasn’t the procedure itself that was more the position I had to put my arm in for them to do the procedure”.

Participant 7

Some participants reported that the position they were required to keep their arm in was very uncomfortable and this was the source of their procedural pain as opposed to the sensation of being injected. Participants reported that they had to maintain their arm in a fixed externally rotated position and that this was aggravating for them given the underlying restriction in their shoulder joint. This demonstrates that technical aspects of treatment delivery were sometimes associated with participant discomfort and pain.

Some participants found the delivery of the injection to the glenohumeral joint to be extremely painful.

“It [the hydrodistension] was just, um, just like someone, like pressure, a real lot of pressure your body couldn’t sustain. You just wanted to fight it off. It was just pressure, real deep pressure”.

Participant 9

“They injected local anaesthetic, and then they injected steroid and then they started to pump it up with the saline. Now he said ‘when we do the saline go to where your absolute maximum tolerance is’… At the end of it I, I’d nearly, I nearly passed out… during the procedure I pushed myself through the pain to a point where I just thought it was gonna explode”.

Participant 8

Some participants reported extreme levels of pain associated with the hydrodistension treatment. These extreme pain levels were associated with a sensation of pressure in the arm. The articulation of the sensation of pain as pressure likely reflects the participants underpinning conceptualisation of frozen shoulder as a condition associated with tightening of the shoulder joint and the proposed effects of hydrodistension to release this. This conceptualisation appears to lead some participants to try to push through the pain sensation as they conceptualise the sense of pressure as their shoulder releasing and thus being of long-term benefit to them.

In summary participants reported a range of experiences associated with hydrodistension of their shoulders ranging from pain free procedures, to positional pain, to extreme pain and sensations of deep pressure, underscoring that there is no single description of having the procedure that adequately captures all these experiences.

#### Acceptability of hydrodistension

The acceptability of hydrodistension among participants was influenced by a number of factors.

“I was anxious about it [the hydrodistension] because I was told it would be, I was told prior to expect that it would be an uncomfortable procedure to go through”.

Participant 12

Some participants reported feeling anxious about the hydrodistension procedure, due to their expectation that the procedure would be painful. While this evoked anxiety was unpleasant it was not a significant barrier to accessing the treatment.

“The procedure was exactly as described and because I was provided with written information on what to expect and it was… things did happen as I expected”.

Participant 13

Participants who received detailed information about the hydrodistension procedure prior to the treatment found this helpful. It allowed them to conceptualise the experience and develop realistic expectations of what the procedure would be like.

“I wasn’t quite prepared for the immediate pain. That was quite a shock cause I’m really good with pain normally, but I thought this is hurting”.

Participant 14

“I think if somebody suggests the hydrodistension to that person that they fully look into it before you go into the radiology room because…I thought it was just like a steroid injection…Mentally I wasn’t prepared no. I think if someone would have sent you a video or to show you what the procedure was, I wouldn’t of had it”.

Participant 9

Participants who had not received information about the procedure beforehand reported almost the opposite experience. These participants reported being shocked at the level of pain associated with the injection and reported finding the experience distressing. One participant reflected that they would not have gone through with the treatment if they had known what it was going to be like suggesting that the procedure has the potential to be very distressing without adequate mental preparation. There appears to be a link between the provision of information about the procedure, adequacy of self preparation, and the ultimate acceptability of the procedure to the individual.

Some participants reported that they were willing to tolerate significant discomfort during the procedure.

“I really did push myself to…I could have wimped out at less, but I wanted it to work. Because when they explained that the anaesthetic is to is to relieve the pain so you can move, the steroid is to reduce the inflammation so you can move and the saline is there to expand the bag or the joint so you can move, so it was in my best interest to do that yeah”.

Participant 8

The motivation for participants to experience pain and discomfort during the procedure appears to stem from the participants understanding of their condition, and the proposed benefits of hydrodistension treatment on their condition. There was a rationalisation that the pain experienced in the procedure was as a result of mechanical expansion of the joint and thus this was constructed as a desirable component of the treatment.

In summary the acceptability of hydrodistension was influenced by participant anxiety about the treatment, the quality of preprocedural information they received, and perceptions of treatment mechanisms. Participants who felt well informed and had rationalised the purpose of the treatment were much more likely to find the procedure acceptable, even in the presence of significant procedural discomfort, than those who received limited or no information.

#### Theme 2: ‘Physiotherapy after hydrodistension’

‘Physiotherapy after hydrodistension’ describes how participants accessed physiotherapy after their hydrodistension and what factors influenced their decision to participate with this process.

#### Physiotherapy after hydrodistension: access to physiotherapy

Participants reported seeing a physiotherapist very quickly after having their hydrodistension procedure.

“it’s much better to see him [the physiotherapist] on the day and to yes, it’s done there and then. To save time and yes, to know what the exercises, new exercises are needed to do straight away as a good, good thing, yeah”.

Participant 13

Participants reported that quick access to the physiotherapist was valuable and appreciated the immediacy of the guidance provided. This allowed participants to begin their post procedural rehabilitation promptly, receive guidance on recommended exercises and set expectations for their recovery.

“Physiotherapy. I was given the usual list… printed list of different exercises to do and that was a bit of an information overload”.

Participant 2

On the other hand some participants felt that seeing a physiotherapist so soon after the procedure was a bit overwhelming. One participant described the list of exercises prescribed on the first appointment with the physiotherapist as information overload, indicating that they likely found the volume of exercise prescribed challenging to deal with.

Participants reported that they saw their physiotherapists on a face to face basis.

“I do but I think a lot of it [physiotherapy] could be done over like Skype or whatever or a facetime type of thing… because it’s like 25 miles for me and sometimes I’m in there for like five minutes”.

Participant 5

A number of participants expressed an interest in receiving some or all of their physiotherapy virtually. They reported that this would be acceptable to them and reported this would likely be associated with a number of benefits including reducing the time burden of their rehabilitation, preventing unnecessary commuting, and increased convenience.

In summary most participants appreciated the immediacy of the physiotherapy they received after their hydrodistension and found this useful in terms of early commencement of rehabilitation and goal setting. For some the proximity of their hydrodistension and physiotherapy appointments was a little overwhelming and likely associated with a reduced engagement with the physiotherapy. Participants expressed a desire for virtual or hybrid alternatives to traditional face to face appointments.

#### Adherence with physiotherapy

Participants reported that they adhered with their prescribed exercises after their hydrodistension.

“She [the physiotherapist] said I’ve got the mobility that I’ve not really got the strength back yet, so that’s what I’m working on when I’m doing those like resistance exercises, I can feel that I haven’t got quite the same strength in the affected shoulder, but I can feel that there is an improvement being made there, so that’s reassuring all the time”.

Participant 12

Participants reported that they were motivated to improve their shoulder function through physiotherapy exercise. Participants expressed that their rationale for proceeding with hydrodistension was to improve the mobility in their shoulder and while this featured as a goal for physiotherapy participants also expressed a desire to progress other facets of their shoulder function including strength. This suggests that physiotherapy brings unique benefits beyond an enhancement of recovery of range of motion after hydrodistension.

“I’ve been to the physio obviously… so they’ve given me some more advanced exercises and when, when I’ve done those, it feels at its best actually to be honest”.

Participant 5

Participants report that performing exercises was linked to sense of wellbeing with their shoulder and this provided a positive feedback loop that enhanced adherence to their prescribed exercises.

“It is just having to constantly move it [the shoulder], to get the benefit out of it [the hydrodistension] you have to constantly push it, so you get the aches and the pains rather than the pain as a result of the injection”.

Participant 4

Some participants reported aches and pains as a result of performing exercises, but they continued to do so intimating that this was part of the process of recovery. They suggested that constant movement was required in order to maintain the benefit of the hydrodistension treatment and thus remained motivated to continue with exercise to derive the expected benefits.

Overall participants demonstrated various motivations for adhering to physiotherapy after their hydrodistension. These included a desire to improve shoulder function, regain strength, and experience a sense of wellbeing with their arm. Some of these motivations exceeded their original rationale for proceeding with the hydrodistension suggesting that physiotherapy had a role in meeting needs not met by the hydrodistension procedure alone. Participants expressed a belief in the long-term benefits of exercise and were therefore willing to undertake exercise despite the potential for additional discomfort.

#### Theme 3: ‘Outcome of hydrodistension’

In this theme participants described side effects they experienced after having hydrodistension, the trajectory of their recovery after treatment, and how they thought about persisting symptoms and the need for any further treatment.

#### Side effects

The treatment was generally well tolerated and not all participants reported side effects from having hydrodistension. For those who did report side effects the most common of these was a transient increase in pain.

“It [the shoulder] suddenly became really, really painful, erm, but that lasted, again, only sort of that four to six hour period that lasted and then it was ok”.

Participant 7

Participants who experienced this transient increase in pain often reported that the pain sensation was quite intense but that it lasted for a relatively short period of time, often from a few hours to a few days. Once the transient increase in pain had passed, participants became more aware of the outcome of the treatment.

Participants reflected on the information provided about this potential side effect.

“I had tonnes of information, and I was expecting to be in quite a bit of pain from like the next day onwards and I have to say I wasn’t”.

Participant 10

“A lot more [pain] than expected yeah. It says on the information leaflet that something about a little bit of stiffness or whatever, so I think that needs maybe rewording”.

Participant 5

Participants had varying expectations regarding what any post procedural pain might be like, and this was based upon information they had received prior to having the procedure. Participants reflected that their lived experiences of post procedural pain was quite different from these expectations. One participant felt that the information leaflet provided didn’t align with their experiences and would benefit from review.

Both participants with type 1 diabetes reported blood sugar dysregulation following their hydrodistension. One of these participants reported that this was easy to manage.

“They [blood sugars] were ok, so I just have my pump, so I just did an increase of 50% insulin for 24 hours yeah so. So that was fine”.

Participant 5

The other participant reported significant instability of their sugars that was extremely difficult to manage and get under control.

“I mean they always do say, cause I’ve had the shoulder steroid, I’ve had the hand steroids, and they always say it might affect your diabetes and normally it’s just a little blip, but this was a big, big, big volcano…I just couldn’t get enough insulin on board and I was ketotic and I did think about going to the hospital”.

Participant 14

The participant reported a familiarity with injected corticosteroids for musculoskeletal health problems and acknowledged that blood sugar dysregulation was a known complication of this, however their previous experiences of this had been as a minor, trivial occurrence. On this occasion the participant indicated that they were almost overwhelmed by the severity of the blood sugar dysregulation and struggled to get this under control.

“I think it is the knowledge having the knowledge there, isn’t it? Then you, you’re aware and you can do something about it, but it was that surprise. It’s sort of, you know I’m having double insulin. I’ll just change everything, nothing. I’ll just double it. Triple it. Nope, it’s not playing”.

Participant 14

The participant reported that they were surprised and under prepared for the level of blood sugar dysregulation following hydrodistension, and that a lack of preparation and plan led them to feel they we’re inadequately equipped to manage the situation. The participant conveyed a sense of fear and being overwhelmed by not being able to immediately control their sugars, although ultimately did not need hospital admission and sugar levels did eventually return to normal.

Participants generally tolerated hydrodistension well, but some participants experienced transient post procedural pain and those with type 1 diabetes some challenges relating to blood sugar dysregulation. Participants emphasised the differences between their expectations of side effects and the reality of them as well as the need to be adequately prepared particularly for the more significant adverse outcomes following the procedure.

#### Trajectory of recovery

Participants reported several different trajectories of recovery following hydrodistension.

“It’s just like switching from a bad shoulder to not having a bad shoulder. It was virtually instant”.

Participant 1

“The pain relief itself was pretty much immediate–I mean, as soon as I stood up off the table, I was like oh, you know that’s better”.

Participant 7

Several participants reported almost instantaneous relief and improvement in frozen shoulder symptoms immediately following their hydrodistension. They described the change as akin to flicking a switch with an immediate recognition of the shoulder moving from one state to another.

“It’s like a miracle”.

Participant 1

The speed and magnitude of the change were so transformative for some participants that they were described as miraculous.

“By the end of the week that I’d had it [the hydrodistension], it was completely different, especially in terms of the pain that [I] had been experienced beforehand. It was phenomenal”.

Participant 10

Another group of participants reported a positive change in symptoms at a slower rate over a few days to a week after having their hydrodistension. These participants noted a substantial improvement in their pain and function over this relatively short time frame. Participants expressed their satisfaction with the pace at which this recovery occurred.

In contrast, some participants in our study described minimal changes to their frozen shoulder symptoms after the procedure.

“I had expected after the injection for the pain to subside and maybe get a greater range of movement and I have got a little bit of extra movement back, but I believe this shoulder is still fused and I’m still suffering quite a considerable bit of pain”.

Participant 6

“I’ve been a bit disappointed in how much more movement I’ve got I suppose… in my mind I’d built it up… I mean well I didn’t expect it to be completely back to normal immediately, but I kind of thought erm It would, I’d have had more movement back by now than I have but er perhaps my expectations were too high”.

Participant 13

Participants expressed that their expectation was that the hydrodistension would cause a significant positive change in their frozen shoulder symptoms in terms of their level of pain and their limited mobility. Participants who didn’t experience these changes following hydrodistension expressed their disappointment in the outcome. Some participants reflected that their initial expectations of the outcome of the procedure had meant that they had expected a better outcome and that this had influenced their frustration with the recovery process.

Regardless of whether symptoms improved or not participants consistently reported that their outcome plateaued within a few weeks of having the procedure.

“The difference I’ve seen since we last talked, obviously, is nothing compared to when we first talked. But I think that’s a good sign. So yeah, a bit boring really. I don’t really have much of an update other than to say it’s almost as if it never happened”.

Participant 10

“Yeah, it’s, the acceleration of improvements [that] has tapered off”.

Participant 2

Those who had perceived a significant initial change in symptoms consistently reported that despite this initial dynamic response to treatment the trajectory of improvement quite quickly slowed and eventually reached a point where further substantial gains were not observed.

To summarise, participants described a range of trajectories in their recovery from hydrodistension, including almost instantaneous improvement, quick changes over a few weeks, or minimal change associated with disappointment. Participants all reported eventual plateaux in recovery.

#### Persisting symptoms and further treatment

Not all participants reported persisting symptoms. Those who did, reported that their arm function was still restricted due to stiffness.

“And even like putting the rucksack on your shoulder, I would have always gone from the left side first and then put my right in, but I can’t do that. And with my seatbelt, I’m not able as easily to kind of reach back over my right shoulder… but it has improved”.

Participant 12

“Yeah, there are times when I try and reach for something and I can’t because my arm won’t go in that, it just won’t go in that direction”.

Participant 4

Participants who reported persisting symptoms highlighted ongoing restriction with activities of daily living due to their shoulder stiffness. Activities such as putting on a backpack or reaching remained challenging. These participants indicated that their desired level of recovery had not been achieved.

For some participants this restriction was significant enough that they felt further treatment of their shoulder was needed.

“But yeah, I do think that I’d like to go down the road to having another one just to see if I can push it that bit further”.

Participant 4

Some participants expressed the desire to proceed with a second hydrodistension, believing that further improvement in their arm function was possible and demonstrating a continued belief in the potential positive effects that hydrodistension could provide.

One participant, whose initial hydrodistension had not been successful, elaborated on why they wished to pursue a second round of treatment.

“I don’t know if it’s an issue with the amount of liquid that’s put into the joint or what it might be, but my hope is that if I can get the injection [second hydrodistension] done then I’ll get that positive outcome again. So, I’ll very much be asking the specialist when I see him to put me forward for that second injection”.

Participant 6

This participant explored the idea that the volume of fluid injected into their shoulder during their initial hydrodistension was insufficient to elicit the required change in their shoulder joint, prompting a desire to proceed with a second hydrodistension. These ideas regarding the therapeutic effect of volume in hydrodistension aligned with the rationale participants gave for choosing to proceed with their initial hydrodistension.

While not all participants reported persisting symptoms those that did particularly reported ongoing functional limitations. For some participants these ongoing symptoms were significant enough that they would consider further treatment, and a second hydrodistension emerged as a consideration. Participants reported sophisticated and complex reasoning regarding their desire to proceed with further hydrodistension, grounded in their understanding of the proposed mechanism of effect of the treatment.

## Discussion

This study explored the lived experience of having a hydrodistension as a treatment for frozen shoulder. Three key themes were identified: ‘preparing for and having a hydrodistension’, ‘physiotherapy after having a hydrodistension’, and ‘outcome of having a hydrodistension’.

Our study found that participants chose to have a hydrodistension treatment due to their understanding of frozen shoulder pathology and the mechanical effects they expected the treatment to have on their shoulder. During shared decision-making health care professionals discussed the treatment in terms of perceived mechanical effect on the shoulder joint and in terms of superiority to low volume corticosteroid injection treatment. Participants found mechanical explanations of how the treatment worked credible and these were often then adopted into their own narrative to conceptualise the treatment and recovery experience. While the mechanical hypothesis for hydrodistension treatment is pervasive there is little evidence to support this narrative. A small single arm cohort study of 18 participants [22] has previously reported a change in capsular volume-pressure ratios following repeated capsular distension. The study concluded that the observed change in capsular volume and flexibility were the result of hydrodistension. However due to the small participant numbers and lack of control arm it is not possible to accept this conclusion with any certainty. In our own study while some participants reported quite dramatic changes in their frozen shoulder symptoms quickly after treatment this did not seem to correlate with the sensation of pressure or stretching during treatment. Further research is required to understand the mechanism of action of hydrodistension treatment as current proposed mechanisms of action are associated with uncertainty. There is uncertainty about whether hydrodistension provides superior clinical outcomes to low volume corticosteroid injection. A recent systematic review [14] identified that the effectiveness of hydrodistension was uncertain based on the results of four randomised controlled trials (RCTs) and that their remains a gap in the evidence to support the treatment. Our study highlights that current shared decision making regarding hydrodistension treatment is sometimes incomplete and does not reflect the known uncertainties about the treatment’s mechanism of action or comparative effectiveness to other treatments. Furthermore, our study highlights that individualised responses to hydrodistension varied widely however this did not seem to be reflected in the information participants had received prior to accessing the treatment, leading some to feel disappointed or frustrated with their outcome. In order to address these shortcomings clinicians should aim to utilise shared decision-making strategies that acknowledge uncertainty [23] to effectively communicate with patients about treatment options. Shared decision making tools and patient information leaflets should be reviewed to ensure they acknowledge a variety of responses and potential outcomes from the treatment. There is a need to address the underlying uncertainty regarding the effectiveness of hydrodistension treatment for frozen shoulder, particularly in comparison to low volume intra-articular corticosteroid injection which is more accessible and cheaper than hydrodistension. Further high-quality prospective research is required to consider the comparative clinical and cost effectiveness of these two treatments.

The experience of undergoing hydrodistension varied considerably but was generally well tolerated. For some this was an almost pain free treatment but for a minority it was an unpleasant experience. Two main reasons for this presented themselves. These were pain associated with procedural position and pain associated with the injection volume. Positional pain was related to the requirement of the arm to be in an externally rotated position to allow access to the anterior glenohumeral joint in the fluoroscopy guided hydrodistension technique. Clinicians could limit procedural pain by choosing to provide the treatment under ultrasound guidance as this allows the arm to rest in a cross-body adduction position as the joint is accessed posteriorly [13]. In terms of injection volume further research is required to understand the mechanism of action in hydrodistension and whether there is a relationship between volume and symptom response. In this current study participants with type 1 diabetes reported that they experienced instability of their blood sugars after receiving the treatment. In one case this was severe despite reported previous good glycaemic control. It is well known that corticosteroid injection can induce an increase in blood glucose in the first 24 hours following treatment [24, 25], however severe dysregulation is rare in patients with previously good glycaemic control [26]. It is not known whether hydrodistension confers additional risk to diabetic patients over and above low volume corticosteroid injection. An elevated risk may be biologically plausible as fluid volume within a joint has been shown to be a factor in systemic uptake in predictive pharmokinetic models of other intra-articularly administered medications [27]. Participants who experienced significant pain during the procedure or who had an adverse event relating to their blood sugars reported that they were insufficiently prepared prior to treatment for these eventualities. Therefore, it is recommended that clinicians adequately discuss the potential harms associated with hydrodistension treatment during the shared decision-making process. Our study suggests that working with type 1 diabetic patients on a blood sugar management plan prior to proceeding with hydrodistension would be good practise and likely reduce the sense of uncertainty should a significant dysregulation occur. Further research is required to establish the risk profile of hydrodistension treatment for patients with type 1 diabetes.

In this study participants were aware of their treatment outcome relatively quickly, usually within the first few days to weeks. This finding is not reflected in the available hydrodistension literature where patient outcomes have typically been collated at 6–12 weeks post intervention [28–31]. Paradoxically our study found that despite this early awareness of outcome participants still placed value on physiotherapy treatment after the procedure and viewed this as important to the recovery process. One reason for this was that some study participants suggested that physiotherapy addressed additional needs that they had with their shoulders beyond the expected effect of the hydrodistension, for example to build up and regain strength. However, it is not known to what extent physiotherapy influences the effect size of injection-based treatments for frozen shoulder. Our results suggest that for participants who experience instantaneous relief or no change in symptoms their outcomes may occur independently of physiotherapy input. Further high-quality prospective research is required to understand whether physiotherapy is associated with additional benefit for patients undergoing injection-based treatments for frozen shoulder.

### Strengths and limitations

This qualitative study provides a comprehensive exploration of the patient experience of hydrodistension for frozen shoulder. The use of a qualitative research design with repeated semi-structured interviews enabled participants to reflect on their experiences and outcomes over time. This meant that a detailed picture of the treatment and recovery process could be captured. We utilised a purposive sampling frame and utilised thematic saturation ensuring that we captured a sample of participants reflective of the frozen shoulder population and that our data reflected a complete exploration of the subject.

The key limitation of the study is that it may have limited generalisability due to the small sample size and that recruitment occurred at only two NHS Trusts in the UK. The study is also limited by the follow up period for the interviews, while we provide an in-depth exploration of the patient experience in the first ten weeks following hydrodistension, longer term outcomes are not explored, limiting the understanding of the long-term impact of the treatment on participants.

## Conclusion

This is the first study to investigate the experiences of people who undergo hydrodistension treatment for frozen shoulder. People with frozen shoulder are likely to choose to have a hydrodistension based upon the idea that the treatment will have a mechanical effect on the shoulder joint and that the relief they will gain from the treatment will be superior to other types of treatment such as low volume corticosteroid injection. Such explanations seem acceptable to people with frozen shoulder but do not capture the known uncertainty regarding the treatment including its mechanism of action and its comparative effectiveness to other treatments. Our study found that the treatment was generally well tolerated but was associated with harm for some people including severe procedural pain and post injection blood sugar dysregulation in type 1 diabetics. Despite the current gap in the evidence base for hydrodistension, our study found that people choose to have the treatment because they think it will improve the mechanical stiffness in their shoulder better than other injection treatments. Our study indicates that people who have a hydrodistension are aware of the outcome of the treatment very quickly afterwards and while post procedural physiotherapy is valued by participants there is uncertainty as to whether this contributes to the outcome of the hydrodistension treatment. We highlight the need for clinicians to engage in shared decision making with patients to acknowledge uncertainties and potential harms associated with hydrodistension. Further high-quality research is required to understand the comparative effectiveness of hydrodistension as a treatment for frozen shoulder, including adverse events, and the benefit of treatment by a physiotherapist after hydrodistension.

## Supporting information

S1 Appendix Topic guide. (PDF)
