# Supplementary material for: Patient experiences of hydrodistension as a treatment for frozen shoulder: A longitudinal qualitative study
Source: PLoS One. 2024 Jun 14;19(6):e0304236. doi: 10.1371/journal.pone.0304236 (PMC11178155; doi:10.1371/journal.pone.0304236)
Supplement: S1 Appendix — (PDF) [file pone.0304236.s001.pdf]

Topic Guide

Research Summary

Frozen shoulder is a painful and debilitating condition which causes long lasting disruption to the physical and mental health of those affected. It can take many years for symptoms to settle and leaves 1 in 5 people with persisting pain or joint stiffness.

Traditional treatments are only partially successful at managing the symptoms so there is interest in finding new ways of treating the problem. One new treatment that is now being offered frequently for the problem is ‘hydrodistension’. This is an injection into the shoulder of pain relieving and inflammation relieving drugs with a volume of sterile water to stretch the joint lining.

Because this treatment is quite new little is known about what it is like to have the treatment, how quickly people recover, and what impact people think it has on their frozen shoulder and quality of life.

This research project has been designed to find out answers to these questions by interviewing people who have this treatment for frozen shoulder. The people who take part in this research will be interviewed twice. Once at 2-4 weeks after treatment to find out why they chose to have the treatment, what they imagined it would be like before the treatment??, what it was like having the treatment, and the progress of their recovery. The second interview will take place at 8-12 weeks after the treatment, and we will be wanting to find out about how their recovery is going and what impact the treatment has had on quality of life since the first interview.

We hope that our research project will give clinicians and patients a better idea about what having this treatment is like so they can make informed decisions about treatment together.

Patient experiences of hydrodistension for frozen shoulder

Interview 1 (2-4 weeks after treatment)

Topic Areas

Life with frozen shoulder
Choosing to have a hydrodistension
Expectations of the treatment
What is it like to have a hydrodistension?
What is the initial recovery from hydrodistension like?
What impact has the treatment had on their life with frozen shoulder

Questions:

Can you start by telling me more about the effects of your frozen shoulder and any previous treatments that you have had?
How it started? What the pain is like and limitation of ROM? How was it diagnosed? What treatment received? Impact on family life, work, hobbies, health, mental health, sleep. How have you been coping?

You've recently had a hydrodistension for your frozen shoulder, can you please tell me how that came about, what information you were given beforehand and your reaction to that information?
How was the treatment offered? How was the treatment portrayed by the clinician? Was it explained how it may help? Was it explained how it may feel? Was recovery discussed? Was shared decision making undertaken or was it more paternalistic? What were their thoughts about the treatment when it was offered?

So now I'd be really interested to hear about what it was like to have the treatment.
What happened on the day? Did they feel prepared? Were they given additional information about the treatment? How did they feel? What was the treatment like? Long? Short? Painful? Comfortable? Vulnerable? In control?

Patient experiences of hydrodistension for frozen shoulder

How do you feel your shoulder is recovering from the treatment?
Immediate aftermath- discomfort,pain, need for analgesics? Symptoms worse, same, better? Thoughts/feelings about experience. Return to day to day life- family life, work, hobbies. How viewed normal activities after treatment- no problem? Fearful? What were their expectations of recovery? Any guidance? Any input from physiotherapy? Exercises?

Has there been any impact on your frozen shoulder symptoms?
How has their pain been? How is the movement in the arm? How are they sleeping How do they feel generally physically and mentally?

Is there anything else you want to tell me, that you think I should know?

Thank the participant for their time.

Patient experiences of hydrodistension for frozen shoulder

Interview 2 (8-10 weeks after treatment)

Topic Areas

Ongoing recovery
Impact of the treatment on their condition and life
Expectations for the future

Questions:

How has your shoulder recovery progressed since we last spoke?
Fully recovered? Still recovering? Day to day life? Work? Physical health? Mental health? Any ongoing treatments (physio, medication) further healthcare consultation?

It's been a few months now since you had your treatment, what do you think the impact has been?
Have things been better, isq, worse? Has it been worthwhile and if so why? Transformational? let down?

What do you think the future holds for you and your shoulder?
Do they feel recovered? Will they be seeking further intervention? Optimistic? Pessimistic?

NHS
York and Scarborough Teaching Hospitals
NHS Foundation Trust

**Is there anything else you want to tell me, that you think I should know?**<br>Would you recommend this treatment to others with a frozen shoulder?
